# Neurodevelopmental Outcomes in Newborns with Congenital Gastrointestinal Atresias

**DOI:** 10.3390/children12091153

**Published:** 2025-08-29

**Authors:** Duygu Tuncel, Senay Guven Baysal, Tulin Oztaş, Nilüfer Okur

**Affiliations:** 1 Department of Pediatrics, Division of Neonatology, SBU Gazi Yasargil Training and Research Hospital, Diyarbakır 21010, Turkey; 2 Department of Pediatrics, Division of Developmental Pediatrics, SBU Gazi Yasargil Training and Research Hospital, Diyarbakır 21010, Turkey; senay177@yahoo.com; 3 Department of Pediatric Surgery, SBU Gazi Yasargil Training and Research Hospital, Diyarbakır 21010, Turkey; tulinoztas@hotmail.com

**Keywords:** neurodevelopment, Bayley III scale, gastrointestinal malformations, neonatal surgery

## Abstract

Background: The population affected by gastrointestinal atresias and neurodevelopmental outcomes has not been well studied. Current evidence suggests that damage to the central nervous system is important in congenital gastrointestinal malformations. This study aims to understand the effects of gastrointestinal atresias on neurodevelopmental outcomes in our patient group. Methods: This cross-sectional, population-based study examined patients with congenital gastrointestinal atresias who were admitted to the neonatal intensive care unit and underwent gastrointestinal surgery. The Bayley III scale was administered to 32 patients aged 7–42 months. Results: Thirty-two patients with gastrointestinal atresia were included in the study. Eighteen (56.2%) of the patients were male. The median gestational age was 37 weeks (range 25–39 weeks) and the median birthweight was 2700 g (range 700–3800 g). A Bayley III evaluation was performed at a median age of 13.7 months (range 7–41 months). The cognitive, motor, and language composite scores were 90, 86, and 89, respectively. The motor score was lower than the cognitive and language scores. No statistical difference was found between low scores, gender and stoma presence in all three neurodevelopmental categories (*p* < 0.05). Conclusion: Patients with congenital gastrointestinal malformations are reported in the literature to have lower motor and language development scores. In our study, lower cognitive and language scores were observed in only one patient, whereas motor delay was more prevalent in the study population. The close neurodevelopmental follow-up of infants with gastrointestinal atresies may improve the quality of life.

## 1. Introduction

Congenital gastrointestinal atresias affect 2.5–3 out of every 10,000 neonates per year [1]. As the perioperative care of these patients has improved, interest in long-term mortality and morbidity has increased. Traditionally, long-term follow-up of patients with congenital gastrointestinal atresias has focused on anthropometric measurements and mechanical growth indicators, such as short bowel syndrome [2]. While these parameters provide important insights into physical well-being, our understanding of other factors impacting neurodevelopmental outcomes, such as genetic predisposition, concurrent systemic anomalies, early surgical intervention, and exposure to hypoxia, remains incomplete [3].

The population with gastrointestinal atresias and neurodevelopmental outcomes has not been well studied. Understanding how early surgical intervention, combined with genetic predisposition, influences neurodevelopmental outcomes in these patients is critical to improve their long-term quality of life. Current evidence suggests that damage to the central nervous system is important in congenital gastrointestinal anomalies. Newborn babies have a developing nervous system. Studies have identified several factors that influence poor neurodevelopmental outcomes, such as prenatal exposure to maternal medications, low birth weight and prematurity, any haemodynamic and respiratory changes, and anaesthetic and surgical procedures [3,4,5,6,7,8]. Inadequate nutritional status is also important. Intestinal ischaemia–reperfusion may trigger a neuroinflammatory response via the gut–brain axis. A recent study has shown the relationship between ischaemic intestinal injury and central nervous system damage in mice [9]. In this perspective, neurodevelopmental follow-up in this population remains of high interest.

The Bayley Scales of Infant and Toddler Development, Third Edition (PsychCorp-Harcourt, Brace, & Co, San Antonio, TX, USA) is the most widely used neurodevelopmental Assesment tool. In recent years, the Bayley Scales have been used in many areas, particularly in hypoxic ischaemic encephalopathy and preterm infants [9,10].

This study aims to focus on the neurodevelopmental status of infants with congenital gastrointestinal atresias.

## 2. Material and Methods

### 2.1. Study Design

This is a cross-sectional population-based study conducted at the Neonatal Intensive Care Unit (NICU) of SBU Gazi Yaşargil Training and Research Hospital, Diyarbakır, Turkey. The study included newborn infants with congenital gastrointestinal atresias who underwent gastrointestinal surgery. The study period was between January 2020 and December 2023. The study protocol was approved by the Institutional Ethics Committee (Protocol number: 2024/186, Date: 12 January 2024) and carried out in accordance with the principles of the Declaration of Helsinki (1964 and its later amendments). Written informed consent was obtained from the parents of all participants.

**Figure 1:**
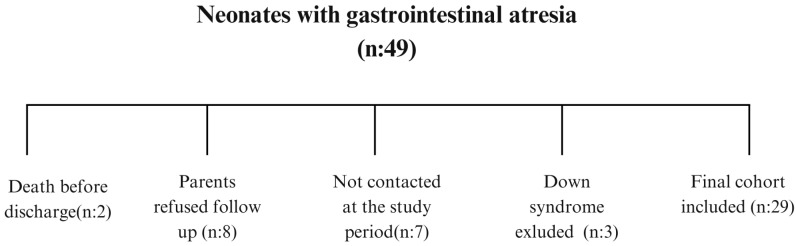
Study flow diagram.

A total of 49 neonates diagnosed with congenital gastrointestinal atresias and operated in the neonatal period were initially identified. Two infants died before hospital discharge, seven families could not be contacted during follow-up, and eight families declined participation. Three neonates with Down syndrome were excluded from the main analysis due to the independent neurodevelopmental effects of chromosomal anomalies. Thus, the final study cohort consisted of 29 infants (Figure 1).

Inclusion Criteria

- Neonates with intestinal atresia admitted to the NICU in the immediate preoperative and postoperative periods.
- Underwent gastrointestinal surgery during the neonatal period.
- Survived to discharge and continued follow-up in our institution.
- Families who provided written informed consent to participate in neurodevelopmental assessment.

Exclusion Criteria

- Patients who died before discharge.
- Patients whose families refuse follow-up.
- Patients who could not be contacted during follow-up.

Demographic and perinatal variables were obtained from hospital records. Perinatal, clinical and surgical variables: type of atresia (duodenal, ileal, gastric, anal), antenatal or postnatal day of diagnosis, gestational age and weight, postnatal day of operation, number of surgical interventions, presence of stoma, anaesthesia exposure, duration of mechanical ventilation, duration of total parenteral nutrition (TPN), time to initiation of enteral feeding, time to achieve full enteral feeding, number of sepsis episodes, length of NICU stay.

**Figure 2:**
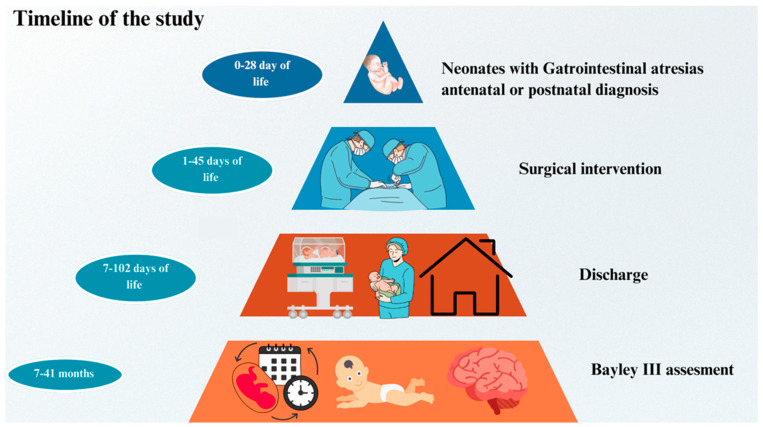
Timeline of study.

Patients who were monitored in the neonatal intensive care unit and underwent surgery due to congenital intestinal atresia were called for developmental paediatrics follow-up after discharge. The patients were monitored at the developmental paediatrics outpatient clinic by a developmental paediatrics specialist (Figure 2).

### 2.2. Assessment of Neurodevelopmental Outcome

The Bayley Scales of Infant and Toddler Development, Third Edition (Bayley III is one of the world’s most widely used instruments for assessing developmental func-tioning in infants and children aged 1–42 months, with well-documented construct validity.

It is considered the gold standard assessment tool for evaluation and research. Comprising five domains—cognitive, receptive language, expressive language, fine motor and gross motor—the Bayley III shows reliability with coefficients of 0.91, 0.86, and 0.87 in the gross motor, cognitive, and receptive language domains, respectively. Each section of the scale can be used independently, allowing for a comprehensive assessment of each developmental domain when assessed as a whole. A systematic review examining the impact of early intervention on motor development highlights the Bayley Scale as the most commonly used method for measuring neuromotor and developmental outcomes [9,10,11]. The structure of the Bayley III provides valuable insight into early development and enhances our ability to identify specific developmental disabilities. From a research perspective, it facilitates a better understanding of early development in high-risk groups and provides more sensitive results for clinical trials [12].

In our study, patients aged 7–42 months who were operated on in the neonatal intensive care unit of our hospital, and whose follow-up was continued in our hospital, were evaluated by a developmental paediatrician. The developmental results of the patients were interpreted as ‘impairment’ for scores between 70 and 84 and ‘severe impairment’ for a score below 70. The neurodevelopmental evaluation of the patients was analysed according to their age ranges.

### 2.3. Statistical Analysis

All statistical analyses were performed using SPSS (IBM SPSS Statistics, Chicago, IL, USA, version 22) and complementary analyses were conducted in R. Descriptive statistics were presented as numbers and percentages for categorical variables, and as medians with interquartile ranges (IQR, 25th–75th percentiles) for continuous variables without normal distribution.

Comparisons of categorical variables between groups were performed using the Chi-square test (Pearson Chi-square). For continuous variables, the Mann–Whitney U test was used in case of non-normal distributions. In addition, when comparing demographic characteristics between the initial cohort (*n* = 32) and the assessed cohort (*n* = 29), independent samples ANOVA was performed where appropriate.

To further explore the structure of the dataset, an Exploratory Principal Component Analysis (PCA) with simple orthogonal (varimax) rotation was conducted. The scree plot was inspected to determine the number of relevant components, and pairwise scatter plots of PC1 vs. PC2, PC3 vs. PC4, and PC5 vs. PC6 were generated to identify potential outliers in the multivariate space. Correlation loadings of original variables with principal components were calculated, and loadings > 0.3 were considered significant. If potential outliers were detected, PCA was repeated after their exclusion to test robustness.

Additionally, a Factor Analysis (FA) was applied to assess the latent factor structure underlying the clinical and perinatal variables, providing complementary insights to PCA.

Correlation analyses were performed using the Spearman test for non-normally distributed variables. Statistical significance was accepted at a level of *p* < 0.05.

## 3. Results

Data from a total of 32 neonates with congenital gastrointestinal obstruction were analysed. The median gestational age was 37 (30–39) weeks and birth weight was 2700 (700–3800) g. Duodenal atresia was the most frequently diagnosed condition (43.75%). Other diagnoses, in order of frequency, were ileal atresia (37.5%), gastric atresia (6.25%), and anal atresia. Three infants with Down syndrome were described separately and excluded from primary analyses to avoid syndrome-related confounding. Diagnosis distribution in this subgroup was duodenal atresia 2 (66.7%) and ileal atresia 1 (33.3%) (Table 1).

**Figure 3:**
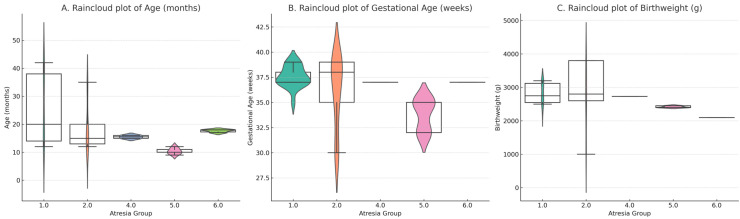
Raincloud plots demonstrating the age at assessment, birth weight, and gestational age (**A**) Age at assessment (months). (**B**) Gestational age at birth (weeks). (**C**) Birthweight (g). Each panel illustrates the distribution of values across different atresia groups using raincloud plots (violin + boxplot + jittered raw data). Median, interquartile range, and 95% confidence intervals are shown.

Demographic and perioperative parameters were compared between all infants (*n* = 32) and the primary analysis set (*n* = 29), no statistically significant differences were detected. The median age at which the patients underwent Bayley III evaluation was 14.5 months (range: 7–42 months). Raincloud plots demonstrating the age at assessment, birth weight, and gestational age are presented in Figure 3. Specifically, male sex was 56.3% vs. 58.6% (*p* = 1.00); median birthweight 2700 g (min–max: 700–3800) vs. 2600 g (min–max: 2400–3120) (*p* = 0.96); gestational age 37 weeks (min–max: 25–39) vs. 37 (min–max: 35–38) (*p* ≈ 0.94); postnatal day of diagnosis 1 (min–max: 1–44) vs. 1 (min–max: 1–4) (*p* = 0.91); day of surgery 3 (min–max: 1–45) vs. 3 (min–max: 2–6) (*p* = 0.93); duration of total parenteral nutrition 14 (min–max: 2–56) vs. 14 (min–max: 10–23) (*p* = 0.96); number of operations 1 (min–max: 1–3) vs. 1 (min–max: 1–1) (*p* = 0.83); number of sepsis episodes 1 (min–max: 1–4) vs. 1 (min–max: 1–3) (*p* = 0.89); length of hospitalisation 27 days (min–max: 7–102) vs. 25 (min–max: 16–44) (*p* = 0.93); Bayley III assessment age 13.5 months (min–max: 7–41) vs. 15 (min–max: 12–20) (*p* = 0.61). These findings indicate that excluding infants with DS did not materially alter the baseline profile of the cohort (Table 2).

In the Bayley III assessment, cognitive CS was 90 (min–max: 90–97.59), motor CS was 86 (77.5–94), and language CS was 89 (78.5–100) in all patients. The motor score is lower than the cognitive and language scores. Cognitive and language impairment was found in 37.8% of the patients, with severe delay in 6.8%. Motor impairment was worse in our study group in 51.6% of the patients. Severe motor impairment was found in 6.8% (Table 3).

For all three domains, gestational week and birth weight were similar, and there was no difference between the groups. The time of diagnosis was only late in the group with lower motor scores (*p* < 0.05). Type of feeding, length of hospital stay, and presence of stoma were not associated with low Bayley III scores (*p* < 0.05) (Table 4).

Three infants had Down syndrome as an additional anomaly. When the Bayley III scores of patients with Down syndrome were evaluated, the cognitive composite score was 55 (55–65), the language composite score was 59 (59–79), and the motor-composite score was 46 (46–49).

**Figure 4:**
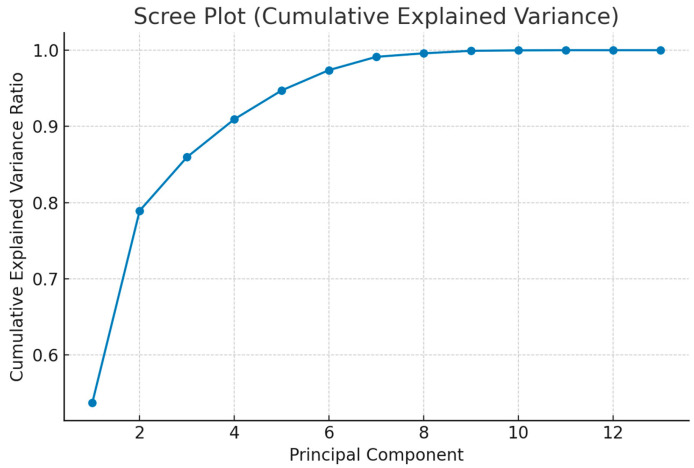
The scree plot shows that variance is largely explained by the first few principal components. This indicates that certain variables exert stronger influences compared to others.

**Figure 5:**
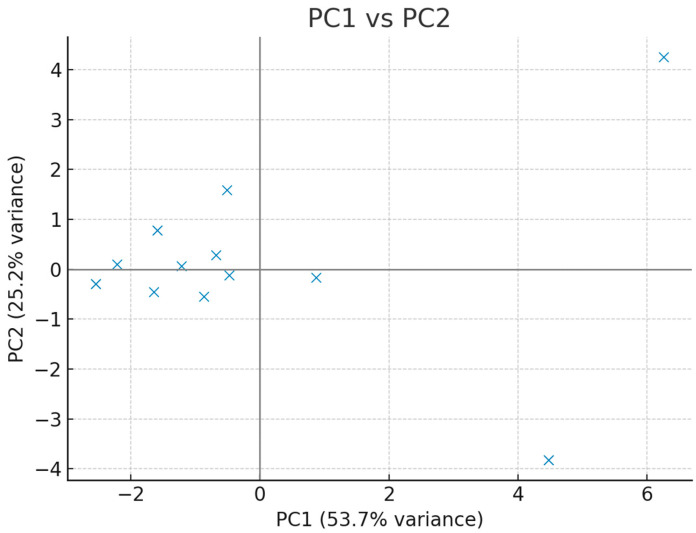
The distribution of participants in the first and second principal component space is shown. No critical outliers were observed.

**Figure 6:**
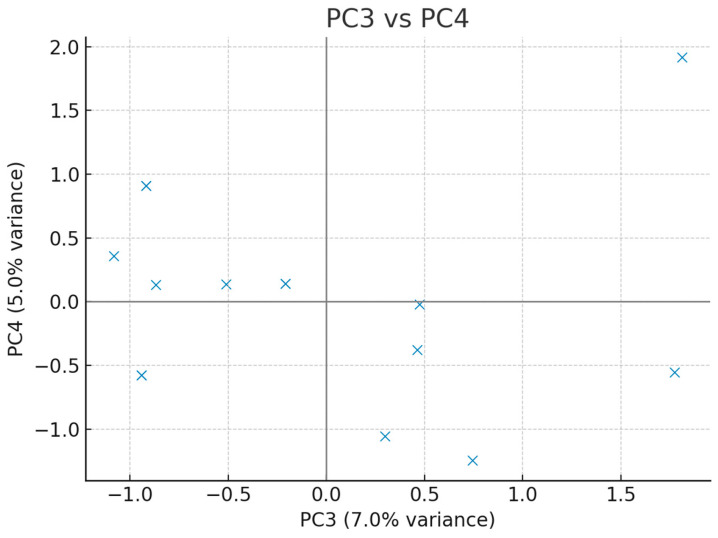
The distribution of participants in the third and fourth principal component space is shown. No critical outliers were observed.

**Figure 7:**
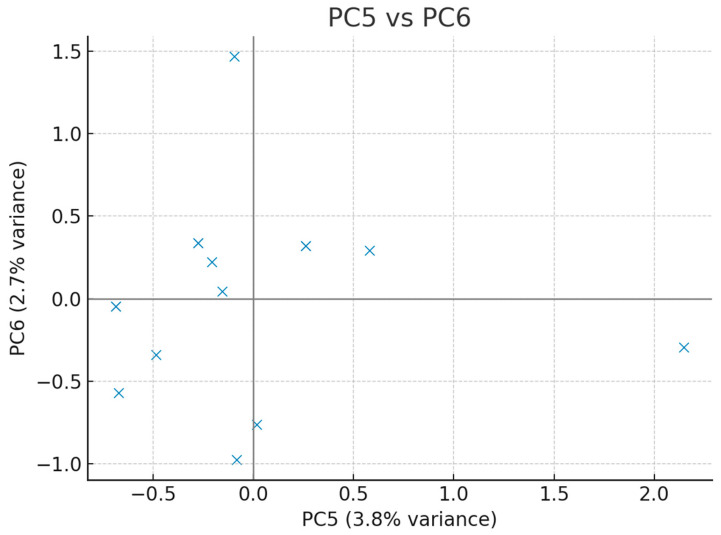
The distribution of participants in the fifth and sixth principal component space is shown. No critical outliers were observed.

Exploratory principal component analysis was conducted to characterise multivariate structure, screen for outliers, and explore collinearity among clinical variables. The scree plot indicated that a limited number of components captured a substantial proportion of variance. Pairwise PC score plots (PC1 × PC2, PC3 × PC4, PC5 × PC6) did not reveal critical outliers. Variable–component correlation (loading) patterns highlighted clinically coherent clusters: PC1 loaded positively on markers of nutritional/hospitalisation burden, whereas PC2 captured peri-diagnostic/timing features with negative contributions from number of operations and stoma. Additional salient loadings included associations of PC4/PC5 with birth parameters and procedure-related variables. Sensitivity checks excluding suspected outliers yielded qualitatively similar patterns (Figure 4, Figure 5, Figure 6 and Figure 7, Table 5).

## 4. Discussion

Children with congenital gastrointestinal atresias are at risk of neurodevelopmental problems. Neonatal surgery for these conditions can lead to suboptimal neurodevelopmental outcomes, with factors such as low birth weight and a prolonged hospital stay increasing the risk. In contrast to the literature, our cohort shows that the motor score is lower than the cognitive and language scores.

Studies have shown that these children may have impaired cognitive, motor, and language abilities [2]. Neonatal surgery for congenital gastrointestinal anomalies has been associated with an increased risk of suboptimal neurodevelopmental outcomes in some studies [2,3,4,5,6,7,8,9,10,11,12,13].

Infants undergoing surgery for abdominal wall defects may experience cognitive and motor delays, with different studies reporting different outcomes. However, the short-term neurodevelopmental outcomes of infants operated on for congenital anomalies, including gastrointestinal anomalies, are generally within the normal range, regardless of the specific malformation. We only included atresia in our study population because infants with other gastrointestinal malformations, such as oesophageal atresia, congenital diaphragmatic hernia, and anterior abdominal wall defects, undergo multiple operations and anaesthesia. For this reason, we believe that each condition should be evaluated individually to more accurately assess neurodevelopmental outcomes.

In our study, only one patient had lower cognitive and language scores, but motor delay tended to be more prevalent in the study population. This may be due to the small size of our sample. Batta et al. found an association between low birth weight and a prolonged hospital stay due to poor neurodevelopment in surgical neonates [13]. Another study investigating gastroschisis and neurodevelopmental outcomes found no such differences at the one-year follow-up. This study found that language scores were poor, particularly in low-birth-weight cases [14]. While many variables were found to be associated with a poor neurological outcome, in our study, only the time of diagnosis was associated with a poor motor outcome. In contrast to other studies, low birth weight and gestational age were not associated with poorer motor, cognitive and language outcomes.

In the study by Doberschuetz et al., 40 patients with gastro-intestinal malformations were assessed with the Bayley II at the age of two years. The patients were found to have delayed verbal skills. It was also reported that exposure to anaesthesia was not associated with neurodevelopmental delay [15].

Although neonatal anaesthesia is thought to be associated with poor neurodevelopment, our study found no difference between the time and reoperation with poor neurodevelopment.

A recently published study assessed patients who had undergone surgery for intestinal atresia at school age. Compared to the control group, children with intestinal atresia had significantly lower mean scores in maths, reading and spelling, indicating potential educational challenges [16]. The study highlights the need for further research and initiatives to improve support systems for children with gastrointestinal malformations, with the aim of enhancing their quality of life and well-being.

**Figure 8:**
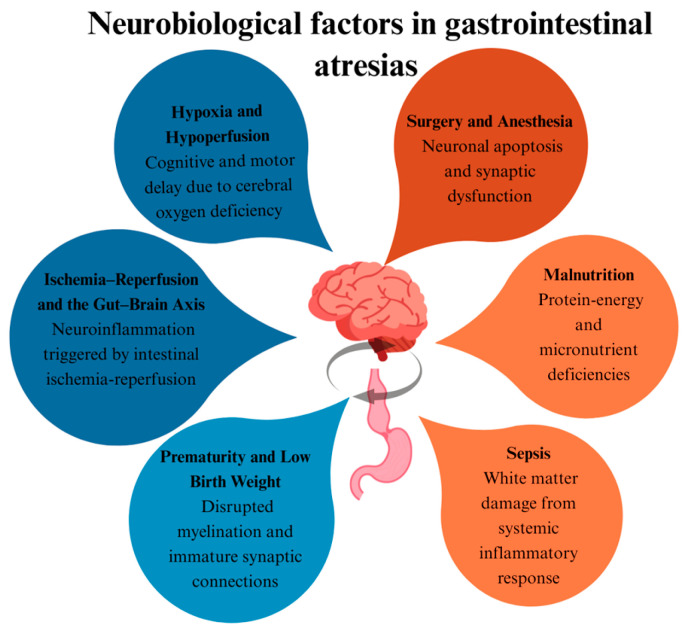
Neurobiological factors affecting neurodevelopment.

The gut–brain axis may influence neurodevelopmental outcomes in newborns with intestinal problems. In addition, factors such as anaesthesia exposure, hypoxia, and sepsis attacks in developing microorganisms may also cause poor neurodevelopmental outcomes in newborns. Supporting these factors with prospective studies will be the subject of future studies [4,5,6,7,8] (Figure 8).

The main limitation of our study is the small sample size.

Explanations regarding the effects of sequential treatment of malformations on neurodevelopmental outcomes cannot be made due to the small sample size and lack of information on various relevant patient characteristics that have a possible impact on neurodevelopmental outcomes. There is no information on birth parameters (e.g., APGAR score, umbilical cord blood pH), need for respiratory support, medications administered, hearing impairments, socioeconomic background, and possible multilingualism in the family.

Although Bayley III is a test developed and controlled in the United States, it is widely used internationally. There are no assessment scales specific to Turkey. The fact that the study was conducted on the Turkish population may affect the results.

We are also aware that it is difficult to say whether the motor delay that we found to be significant in our study was due to the disease or due to anaesthesia and intensive care unit treatments because of such a small patient group and lack of a control group.

However, we think that our study will contribute to the literature in terms of future studies since the data from studies evaluating using the Bayley scale with only intestinal atresia in childhood are limited. In addition, we wanted to draw attention to the need for close neuromotor and physical rehabilitation in children with intestinal atresia and the need for follow-up procedures in children who operated in the neonatal period because of gastrointestinal atresias.

Routine screening and neurodevelopmental follow-up of children with congenital gastrointestinal anomalies is necessary to identify those who may benefit from early intervention services and to improve neurological and motor outcomes.

## Figures and Tables

**Table 1:** X Diagnosis of patients with and without down syndrome.

Patients Excluding Down Syndrome Cases *n* (29)	
**Duodenal atresia, *n* (%)**	14 (43.3)
Ileal atresia, ***n*** (%)	11 (37.9)
Gastric atresia, ***n*** (%)	2 (6.9)
Anal atresia, ***n*** (%)	2 (6.9)
**Patients with Down Syndrome *n* (3)**	
**Duodenal atresia, *n* (%)**	2 (66.7)
Ileal atresia, ***n*** (%)	1 (33.3)

**Table 2:** Perinatal and clinical characteristics of the patients.

	All Patients (*n* = 32)	Patients Without Down Syndrome (*n* = 29)	*p*
**Male, *n* (%)**	18 (56.25)	17 (58.6)	1.00
**Birthweight, g ∗**	2700 (700–3800)	2600 (2400–3120)	0.96
**Gestation week ∗**	37 (25–39)	37 (35–38)	0.94
**Postnatal day of diagnosis ∗**	1 (1–44)	1 (1–4)	0.91
**The day of surgery ∗**	3 (1–45)	3 (2–6)	0.93
**Duration of total parenteral nutrition ∗**	14 (2–56)	14 (10–23)	0.96
**Number of operations ∗**	1 (1–3)	1 (1–1)	0.83
**Duration of enteral feeding ∗**	6 (3–51)	14 (12–28)	0.96
**Number of sepsis attacks ∗**	1 (1–4)	1 (1–3)	0.89
**Duration of hospitalisation, day ∗**	27 (7–102)	25 (16–44)	0.93
**Bayley III assessment age, months ∗**	13.5 (7–41)	15 (12–20)	0.61
**∗ median (IQR 25–75)**			

∗: median.

**Table 3:** Bayley III test evaluation results of patients.

	*n* = 29
**Cognitive composite score**	90 (80–97.5)
**Cognitive score 70–84, *n* (%)**	9 (31)
**Cognitive score < 70, *n* (%)**	2 (6.8)
**Motor composite score**	86 (77.5–94)
**Motor score 70–84, *n* (%)**	13 (44.8)
**Motor score < 70, *n* (%)**	2 (6.8)
**Language composite score**	89 (78.5–100)
**Language score 70–84, *n* (%)**	9 (31)
**Language score < 70, *n* (%)**	2 (6.8)
* median (IQR 25–75)	

* median.

**Table 4:** Factors affecting neurodevelopmental outcomes.

	LCS > 84 (*n* = 16)	LCS ≤ 84 (*n* = 10)	*p*	MCS > 84 (*n* = 12)	MCS ≤ 84 (*n* = 14)	*p*	CCS > 84 (*n* = 16)	CCS ≤ 84 (*n* = 10)	*p*
**Gestational week**	37 (33–37)	37 (27–39)	**0.71**	37 (35–38)	36 (28–37)	**0.36**	37 (31–38)	37 (35.5–38.5)	**0.61**
**Birth weight, g**	2500 (2250–2625)	2825 (2825–3612)	**0.60**	2730 (2450–3120)	2350 (925–2750)	**0.18**	2500 (1700–2960)	2665 (2225–2970)	**0.82**
**Time of diagnosis, day**	1 (1–4.5)	2.5 (1.25–15.75)	**0.33**	0 (0–1)	4.5 (2.8–26)	**0.01**	1 (0–12.5)	2.5 (1.25–3.75)	**0.50**
**Time of operation, day**	3 (1.5–6)	3.5 (2.2–20.5)	**0.71**	2 (1–3)	6 (3.8–30.8)	**0.05**	3 (2–16.5)	3.5 (1.25–3.75)	**0.94**
**Duration of TPN, day**	15 (8.5–25)	27.5 (10–45.75)	**0.71**	14 (0–22)	21 (9.3–45)	**0.62**	21 (12–36)	8.5 (3.25–37)	**0.19**
**Time to start enteral feeding**	7 (4–23.5)	12.5 (12.5–27)	**0.82**	6 (4–16)	12.5 (6–35)	**0.29**	7 (5–30)	5.5 (3.25–15.2)	**0.41**
**Full enteral feeding time**	21 (12–40.5)	29.4 (12.2–48.2)	**0.50**	14 (11–23)	34.5 (13–51)	**0.44**	23 (13–53.5)	13 (4.75)	**0.19**
**Duration of hospitalization, day**	27 (14–52.5)	45 (17.7–94)	**1**	25 (12–44)	44 (16.7–38)	**0.23**	29 (22–64)	16.5 (9.25–56)	**0.26**
**Median (IQR 25–75)**									

**Table 5:** The correlations between the original variables and the new principal components in the Bayley dataset are presented. Loadings greater than 0.3 are marked with an asterisk (*) to highlight critical associations.

	PC1	PC2	PC3	PC4	PC5
**Gestational age, week**	−0.30 *	−0.20	−0.13	−0.23	−0.52 *
**Birthweight, g**	−0.27	−0.24	0.01	−0.46 *	−0.28
**Time of diagnosis, day**	0.24	0.39 *	−0.15	0.07	−0.29
**Time of operation, day**	0.26	0.37 *	−0.18	0.05	−0.32 *
**Number of surgeries ı**	0.23	−0.41 *	−0.08	0.08	−0.20
**Duration of TPN, day**	0.30 *	−0.30	−0.11	−0.22	0.01
**Stoma**	0.14	−0.36 *	0.31 *	0.63 *	−0.42 *
**Time to start enteral feeding**	0.37 *	0.08	−0.11	0.05	−0.10
**Full enteral feeding time, day**	0.35 *	−0.12	−0.16	−0.22	−0.15
**Duration of hospitalization, day**	0.34 *	−0.16	−0.10	−0.33 *	0.05

## Data Availability

The data supporting the findings of this study are not publicly available due to patient privacy and ethical restrictions but are available from the corresponding author upon reasonable request.
